# Systemic Inflammatory Biomarkers as Prognostic Indicators in Metastatic Colorectal Cancer: A Retrospective Study

**DOI:** 10.3390/medicina62071259

**Published:** 2026-06-30

**Authors:** Diana-Ioana Panaite, Simona-Ruxandra Volovat, Madalina Ostafe, Cezara-Ioana Litcanu, Cristian-Constantin Volovat, Maria-Luiza Baean, Ingrid-Andrada Vasilache, Constantin Volovat

**Affiliations:** 1 Grigore T. Popa University of Medicine and Pharmacy, 700115 Iasi, Romaniacvolovat@gmail.com (C.V.); 2 Faculty of Midwifery and Nursing, University of Medicine and Pharmacy “Carol Davila”, 050474 Bucharest, Romania; 3 Faculty of Medicine and Biological Sciences, ‘Ștefan cel Mare’ University, 720229 Suceava, Romania

**Keywords:** metastatic colorectal cancer, inflammation, neutrophil-to-lymphocyte ratio, platelet-to-lymphocyte ratio, C-reactive protein, survival analysis, Cox regression, prognostic biomarkers

## Abstract

*Background and Objectives*: Systemic inflammatory biomarkers have emerged as potential prognostic indicators in metastatic colorectal cancer (mCRC). However, the prognostic robustness of inflammatory indices such as neutrophil-to-lymphocyte ratio (NLR), platelet-to-lymphocyte ratio (PLR), C-reactive protein (CRP), C-reactive protein-to-albumin ratio (CAR), and Glasgow Prognostic Score (GPS) remains incompletely characterized. In this study, we aimed to evaluate the prognostic significance of NLR, PLR, CRP, CAR, and GPS for progression-free survival in metastatic colorectal cancer in a cohort of patients from Romania. *Materials and Methods*: This retrospective observational study included 148 patients diagnosed with mCRC. Inflammatory biomarkers were determined from baseline laboratory parameters. Progression-free survival (PFS) was the primary endpoint. Statistical analyses included correlation testing, Kaplan–Meier survival analysis, Cox proportional hazards regression, Firth penalized Cox regression, restricted cubic spline modeling, time-dependent receiver operating characteristic (ROC) analysis, LASSO penalized regression, multiple imputation, and parsimonious multivariable Cox models adjusted for major clinicopathologic confounders. *Results*: Median PFS was 21 months (95% CI 19–24). In univariable Cox analyses, elevated NLR (HR 1.98, 95% CI 1.11–3.51, *p* = 0.020), PLR (HR 1.89, 95% CI 1.25–2.85, *p* = 0.002), CRP (HR 1.45, 95% CI 1.15–1.83, *p* = 0.002), and CAR (HR 1.44, 95% CI 1.05–1.98, *p* = 0.022) were associated with shorter PFS. Restricted cubic spline analysis demonstrated a significant nonlinear association between NLR and PFS (*p* = 0.0025). After multiple imputation, NLR remained associated with shorter PFS (HR 2.04, 95% CI 1.13–3.68, *p* = 0.018). However, in a multivariable model adjusted for major clinicopathologic confounders, this association was not retained (HR 1.41, 95% CI 0.81–2.43, *p* = 0.221) and time-dependent ROC analyses demonstrated its limited discriminatory performance. *Conclusions*: Although some inflammatory markers were associated with shorter PFS in univariable analyses, the prognostic effect of NLR was attenuated after adjustment and was not consistently confirmed across all analyses.

## 1. Introduction

Colorectal cancer (CRC) remains one of the leading causes of cancer-related mortality worldwide, with a continuously increasing global burden and projected incidence growth through 2040, particularly in low- and middle-income countries and among younger individuals [1,2]. Recent reviews have emphasized the molecular heterogeneity of CRC, the role of tumor subtypes and tumor sidedness, as well as the need for personalized treatment approaches and modern management strategies for advanced and metastatic disease [3,4,5]. In parallel with the refinement of systemic therapies, including chemotherapy, targeted therapies, and immunotherapy, easily accessible blood-based biomarkers have gained increasing importance for prognostic stratification.

Several large studies have comparatively evaluated various systemic inflammatory biomarkers. Leitch et al. demonstrated that an acute-phase response-based score, the modified Glasgow Prognostic Score (mGPS), was a superior predictor of survival compared with cellular components of the inflammatory response, including neutrophils, lymphocytes, monocytes, and platelets, both in patients undergoing curative-intent surgery and in those with unresectable synchronous liver metastases [6].

Subsequently, Dolan et al. compared composite ratios such as NLR (neutrophil-to-lymphocyte ratio), PLR (platelet-to-lymphocyte ratio), LMR (lymphocyte-to-monocyte ratio), and CAR (C-reactive protein-to-albumin ratio) with cumulative scores including mGPS, NLS (neutrophil-lymphocyte score), PLS (platelet-lymphocyte score), LMS (lymphocyte-monocyte score), and NPS (neutrophil-platelet score) in patients undergoing surgery for colon cancer, demonstrating that all retained prognostic value independent of TNM (tumor, node, metastasis) stage [7]. However, cumulative scores based on standard reference intervals were considered simpler and more consistent for clinical use [7].

In a large prospective cohort of patients undergoing curative resection, Lee et al. showed that elevated GPS values (1 or 2), derived from CRP (C-reactive protein) and albumin, were associated with increased postoperative morbidity risk as well as reduced overall and disease-free survival [8]. On the other hand, Park et al. simultaneously analyzed serum cytokines (such as interleukin-8, IL-8) and systemic inflammatory markers (mGPS, NLR, PLR, LMR, PNI- prognostic nutritional index, fibrinogen), demonstrating weak correlations between cytokines and systemic indices but improved prognostic performance when these categories were integrated into a composite classification [9].

Recent interest has expanded toward novel integrative inflammatory indices. Chen et al. introduced the systemic immune-inflammation index (SII), based on the combination of platelets, neutrophils, and lymphocytes, demonstrating in a cohort of 1383 patients that SII was an independent predictor of overall and disease-free survival, with higher ROC area-under-the-curve values than NLR and PLR and superior discrimination across TNM subgroups [10]. Similarly, Xie et al. performed a large-scale comparative analysis of multiple serum inflammatory biomarkers and demonstrated that the inflammatory burden index (IBI) had the highest prognostic predictive accuracy in CRC. Also, elevated IBI values were associated with poorer overall survival, increased short-term complication risk, malnutrition, and recurrence [11].

In younger patients with resectable CRC, Lee et al. demonstrated that inflammatory ratios such as NLR, LMR, and PNI retained prognostic relevance, although the profile of independent prognostic markers differed between patients aged ≤ 50 years and older individuals, suggesting that systemic inflammation contributes to the distinct biological behavior of CRC in younger populations [12]. Specifically for early-onset CRC, Xiang et al. compared seven nutritional and inflammatory biomarkers and identified SII and the geriatric nutrition risk index (GNRI) as the strongest predictors of overall survival, with elevated SII and reduced GNRI associated with significantly worse prognosis [13].

A review focused on metastatic colorectal cancer (mCRC) demonstrated that elevated pre-treatment NLR and PLR were consistently associated with poorer overall survival (OS), disease-free survival (DFS), and progression-free survival (PFS) in patients with metastatic disease. Increased CAR values and reduced albumin-to-globulin ratios were also associated with inferior OS and PFS outcomes [14].

In a large cohort of patients with mCRC receiving palliative treatment, elevated NLR was associated with hazard ratios for PFS ranging between 1.30 and 1.44 across first-, second-, and third-line treatment settings. PLR was identified as a predictor of 6-month PFS, while elevated CRP levels predicted poorer PFS in patients receiving first- and second-line therapy as well as best supportive care [15].

In patients with metastatic colorectal cancer and liver metastases, pre-treatment CRP, GPS, and CAR were identified as independent prognostic factors for overall survival. Elevated levels of these inflammatory biomarkers were associated with significantly shorter survival durations [16].

In addition to these individual indices, combined prognostic models appear to provide more refined risk stratification. Moro-Valdezate et al. developed a prognostic nomogram for 3-year survival using routine inflammatory biomarkers, including neutrophils, lymphocytes, platelets, PNI, and SII, integrated with clinical variables, achieving good predictive performance for cancer-specific and disease-free survival [17]. Similarly, Chen et al. proposed a combined pan-immune-inflammation value–prognostic nutritional index (PIV-PNI) score; higher scores, reflecting intense systemic inflammation and impaired nutritional status, were associated with significantly reduced overall and disease-free survival, while the nomogram based on this score outperformed several traditional staging systems [18]. These results support the concept that composite scores integrating inflammation and nutritional status may more accurately capture the complex interaction between host and tumor biology, as well as the clinical heterogeneity of colorectal cancer progression.

In this study, we aimed to evaluate the prognostic significance of NLR, PLR, CRP, CAR, and GPS for progression-free survival in metastatic colorectal cancer in a cohort of patients from Romania. In addition, we sought to determine optimal biomarker cut-off values using Kaplan–Meier analysis and to test the hypothesis that the relationship between NLR and progression-free survival may demonstrate nonlinear behavior across the biomarker spectrum.

## 2. Materials and Methods

This retrospective cohort study included 148 patients diagnosed with mCRC who underwent clinical diagnosis and follow-up within Victoria Hospital between January 2018 and December 2025. Both synchronous metastatic disease at initial diagnosis and metachronous metastatic recurrence following prior non-metastatic colorectal cancer treatment were included. Metastatic status was established based on multidisciplinary oncologic evaluation and radiologic documentation available in the retrospective clinical records. Clinical, laboratory, and survival data were retrospectively collected from electronic medical records and anonymized prior to statistical analysis.

The study was conducted in accordance with the Declaration of Helsinki. The protocol was approved by the local institutional ethics committees (Victoria Hospital Iasi-2855/15.02.2025; Grigore T. Popa University of Medicine and Pharmacy Iasi-672/12.11.2025), and written informed consent was obtained from all participants prior to data processing and analysis.

Progression-free survival (PFS) was considered the primary endpoint and was defined from the date of initiation of first-line systemic therapy for metastatic disease to the date of first documented progression, with censoring at last available follow-up for patients without progression. OS was defined as the interval between initiation of first-line systemic therapy for metastatic colorectal cancer and death from any cause, with censoring performed at the last available follow-up for surviving patients.

Baseline inflammatory biomarkers were derived from routine laboratory investigations performed prior to treatment initiation or oncological intervention.

The analyzed inflammatory biomarkers included:

- Neutrophil-to-lymphocyte ratio (NLR).
- Platelet-to-lymphocyte ratio (PLR).
- C-reactive protein (CRP).
- C-reactive protein-to-albumin ratio (CAR).
- Glasgow Prognostic Score (GPS).

The biomarkers were operationally defined as follows:

- NLR = Neutrophils/Lymphocytes.
- PLR = Platelets/Lymphocytes.
- CAR = CRP/Albumin.

GPS was calculated using CRP and albumin values according to standard criteria:

- GPS = 0 if CRP ≤ 10 mg/L and albumin ≥ 35 g/L.
- GPS = 1 if either CRP > 10 mg/L or albumin < 35 g/L.
- GPS = 2 if CRP > 10 mg/L and albumin < 35 g/L.

Logarithmic transformation and winsorization procedures were applied to CRP and CAR prior to Cox regression modeling.

Progression-free survival (PFS) was defined as the interval between surgery or treatment initiation and documented radiological or clinical disease progression or death from any cause, whichever occurred first. Patients without progression events were censored at the date of the last available follow-up. OS was defined as the interval between intervention and death from any cause, with censoring performed at the last available follow-up for surviving patients and was considered a secondary outcome.

Descriptive statistics included mean ± standard deviation (SD), median values, and interquartile ranges (IQR). Missing data patterns were additionally evaluated for inflammatory biomarkers and survival-related variables.

Normality testing was performed using the Shapiro–Wilk test. Correlations between inflammatory biomarkers and survival outcomes were evaluated using both Pearson and Spearman correlation coefficients.

Optimal cut-off values for inflammatory biomarkers associated with progression-free survival were determined using receiver operating characteristic (ROC) analysis and the Youden index criterion, followed by false discovery rate (FDR) adjustment for multiple testing. Kaplan–Meier survival analysis and log-rank testing were subsequently used to evaluate survival differences between biomarker-defined groups.

Risk quantification was performed using univariable Cox proportional hazards regression models for progression-free survival, incorporating logarithmically transformed predictors. For CRP and CAR, winsorized transformed variables were additionally analyzed to reduce the influence of extreme values.

Given the limited number of OS events and the risk of sparse-event bias and quasi-complete separation, Firth penalized Cox regression models were used for overall survival analyses.

Variable selection and overfitting reduction were explored using Least Absolute Shrinkage and Selection Operator (LASSO) penalized Cox regression. Potential nonlinear effects of NLR on progression-free survival were investigated using restricted cubic spline (RCS) modeling. Sensitivity analyses using multiple imputation by chained equations (MICE) were additionally performed to evaluate the robustness of the primary findings.

We performed parsimonious multivariable Cox proportional-hazards models adjusted for age, ECOG performance status, metastatic burden, tumor sidedness, and anti-VEGF exposure in order to address major clinicopathologic confounding. Because proportional-hazards violations were observed for ECOG in preliminary analyses, final adjusted models were stratified by ECOG performance status.

Time-dependent receiver operating characteristic (ROC) analysis, and bootstrap internal validation, were further performed to evaluate model discrimination, stability, and predictive performance.

A composite inflammatory score was constructed as an exploratory summary inflammatory index. NLR, PLR, CRP, and CAR were first log-transformed to reduce skewness and subsequently standardized using z-score normalization together with GPS values. The final composite inflammatory score was calculated as the unweighted sum of standardized biomarker values:

Composite inflammatory score = z(log_NLR) + z(log_PLR) + z(log_CRP) + z(log_CAR) + z(GPS)

The association between the composite score and progression-free survival was subsequently evaluated using Cox proportional hazards regression models.

All statistical analyses were conducted using R statistical software (version 4.5.3, R Foundation for Statistical Computing, Vienna, Austria). Two-sided *p*-values < 0.05 were considered statistically significant.

## 3. Results

Among the 148 included patients, 71 (48.0%) presented with synchronous metastatic disease at diagnosis, while 21 (14.2%) developed metachronous metastatic disease during follow-up. In 56 cases (37.8%), metastatic timing could not be retrospectively reconstructed with certainty because of incomplete historical staging documentation.

Liver metastases were the most frequent metastatic site (77.7%), followed by pulmonary (60.8%) and lymph node metastases (57.4%). Multisite metastatic disease was observed in 66.2% of patients. Left-sided primary tumors accounted for 66.2% of cases, whereas right-sided tumors represented 31.1%.

Molecular profiling showed KRAS mutations in 54.7% of patients, NRAS mutations in 23.0%, BRAF mutations in 5.4%, and MSI-H status in 2.7%. All patients received first-line systemic therapy, while 48.6% and 11.5% subsequently received second-line and third-line treatment, respectively. Anti-VEGF exposure was documented in 77.0% of patients and anti-EGFR exposure in 43.2%. Oxaliplatin-based regimens were administered in 74.3% of patients and irinotecan-based regimens in 59.5%.

Kaplan–Meier analysis showed a median progression-free survival of 21 months (95% CI: 19–24) and a median overall survival of 31 months (95% CI: 29–49).

Baseline inflammatory biomarkers demonstrated substantial inter-patient variability across the study cohort (Table 1). Median NLR was 3.20 (IQR 1.80–4.15), while median PLR was 188.00 (IQR 122.96–256.30). Both markers showed relatively broad distributions, particularly PLR, which ranged from 73.45 to 602.90. GPS values were generally low, with a median score of 0 (IQR 0–1), suggesting that most patients did not present with severe combined inflammatory and nutritional impairment at baseline.

CRP and CAR exhibited markedly skewed distributions with considerable dispersion and wide ranges. Median CRP was 5.01 mg/L (IQR 1.63–20.78), whereas the mean CRP value was substantially higher (17.70 ± 28.29), reflecting the influence of extreme inflammatory values in a subset of patients. Similarly, CAR demonstrated a median value of 1.44 (IQR 0.39–4.72), with a wide overall range extending up to 26.99. These findings supported the subsequent use of logarithmic transformation and winsorization procedures during survival modeling.

Albumin levels were comparatively stable, with a median value of 4.11 g/dL (IQR 3.71–4.36). Nevertheless, lower albumin values were observed in some patients, with measurements as low as 1.42 g/dL, indicating the presence of clinically relevant nutritional impairment in part of the cohort.

**Figure 1:**
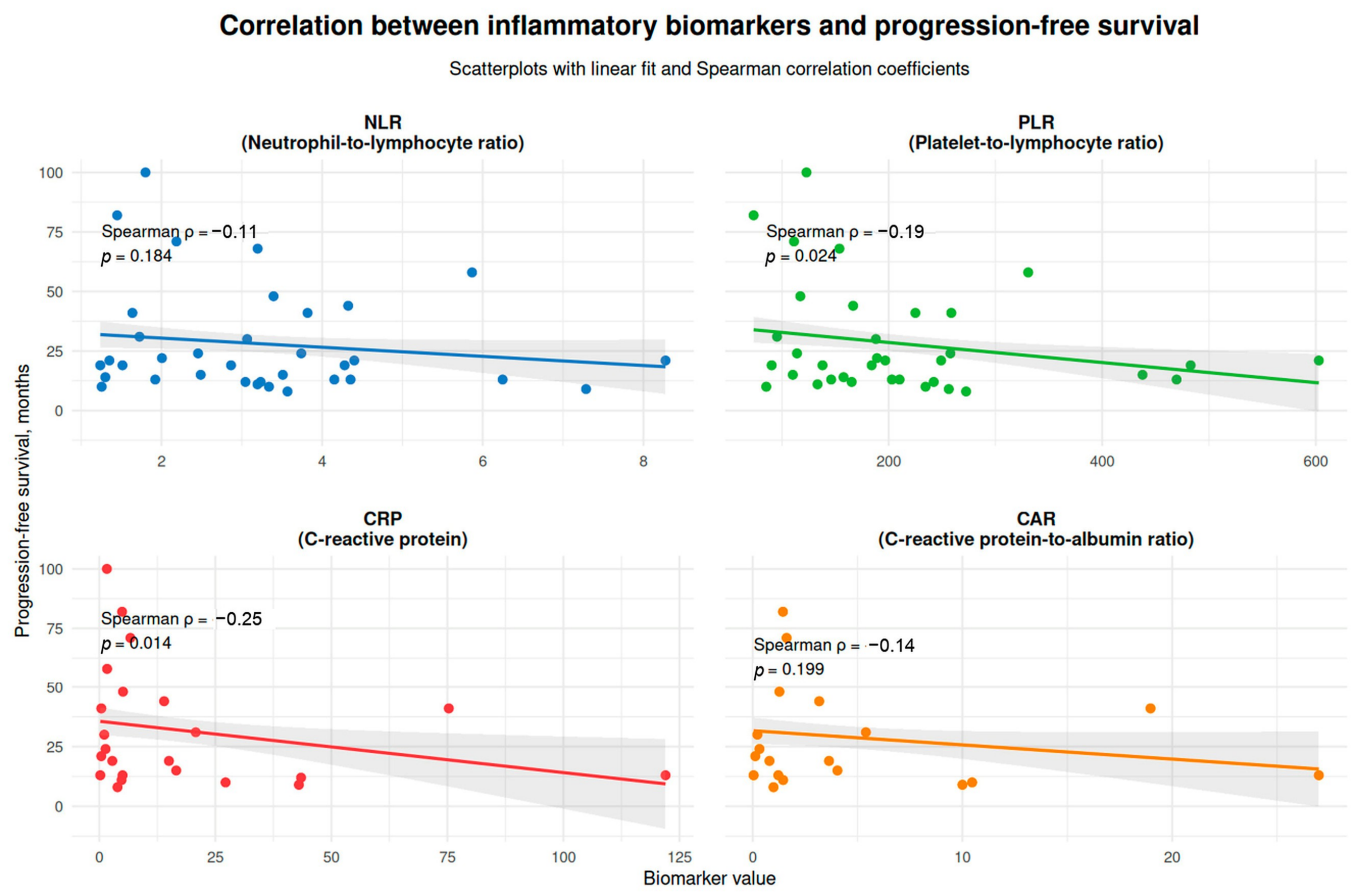
Correlation between inflammatory markers and PFS.

Correlation analyses demonstrated predominantly inverse associations between inflammatory biomarkers and survival outcomes, indicating that higher systemic inflammatory burden tended to be associated with shorter progression-free and overall survival (Table 2 and Figure 1).

The strongest correlations with PFS were observed for CRP and PLR. CRP demonstrated significant inverse correlations with PFS in both Pearson (r = −0.245, *p* = 0.016) and Spearman analyses (ρ = −0.251, *p* = 0.014), while PLR also showed significant negative correlations with PFS using both methods (Pearson r = −0.227, *p* = 0.006; Spearman ρ = −0.185, *p* = 0.024).

NLR demonstrated a weaker inverse association with PFS that did not reach statistical significance. However, NLR showed a significant negative Pearson correlation with OS (r = −0.207, *p* = 0.011), suggesting that elevated NLR values may be associated with shorter overall survival. Similarly, GPS demonstrated significant inverse correlations with OS in both Pearson and Spearman analyses, with higher GPS values associated with poorer survival outcomes.

CAR demonstrated a trend toward inverse correlation with PFS, although statistical significance was not reached in either Pearson or Spearman analyses. No meaningful correlations were identified between CAR and OS.

**Figure 2:**
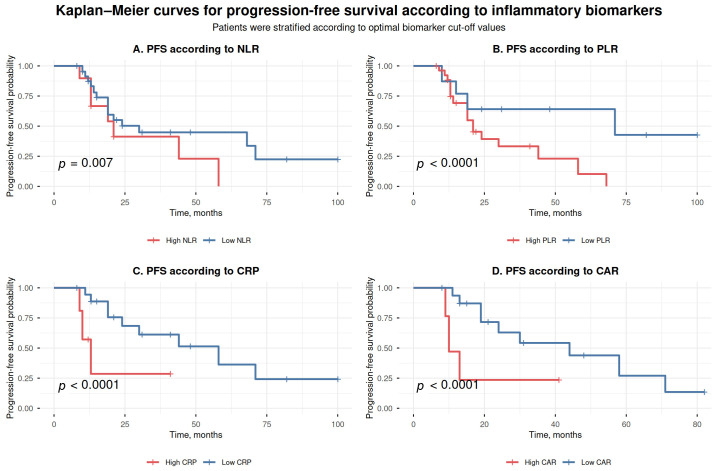
KM curves for PFS according to inflammatory markers.

Kaplan–Meier survival analysis demonstrated significant differences in progression-free survival according to several inflammatory biomarkers (Table 3 and Figure 2). Among the evaluated markers, PLR, CRP, and CAR showed the strongest discriminatory performance, with highly significant log-rank statistics and persistence of significance after false discovery rate adjustment.

Patients with NLR values above the optimal cut-off of 4.15 experienced significantly shorter progression-free survival compared with those below this threshold (χ^2^ = 9.46, *p* = 0.002). Similarly, elevated PLR values above 122.96 were associated with markedly poorer PFS (χ^2^ = 18.64, *p* < 0.001). CRP and CAR demonstrated comparable prognostic separation, with higher values strongly associated with adverse progression-free survival outcomes. In contrast, GPS did not demonstrate significant discrimination for PFS within this cohort.

In univariable Cox regression analysis for progression-free survival, logarithmic transformation and winsorization procedures were applied to address skewed biomarker distributions and the influence of extreme values (Table 4).

Elevated inflammatory biomarkers were generally associated with shorter progression-free survival. Increased NLR remained significantly associated with poorer PFS, with each logarithmic increase corresponding to an approximately twofold increase in progression risk (HR 1.98, 95% CI 1.11–3.51, *p* = 0.020). Similarly, elevated PLR demonstrated a significant association with adverse PFS outcomes (HR 1.89, 95% CI 1.25–2.85, *p* = 0.002).

Among acute-phase inflammatory markers, both CRP and CAR retained significant prognostic value after winsorization and logarithmic transformation. Elevated CRP was associated with a 45% increase in progression risk (HR 1.45, 95% CI 1.15–1.83, *p* = 0.002), while higher CAR values were associated with shorter PFS (HR 1.44, 95% CI 1.05–1.98, *p* = 0.022).

On the other hand, GPS did not demonstrate a statistically significant association with progression-free survival in this cohort, although a trend toward poorer outcomes with increasing GPS values was observed.

**Figure 3:**
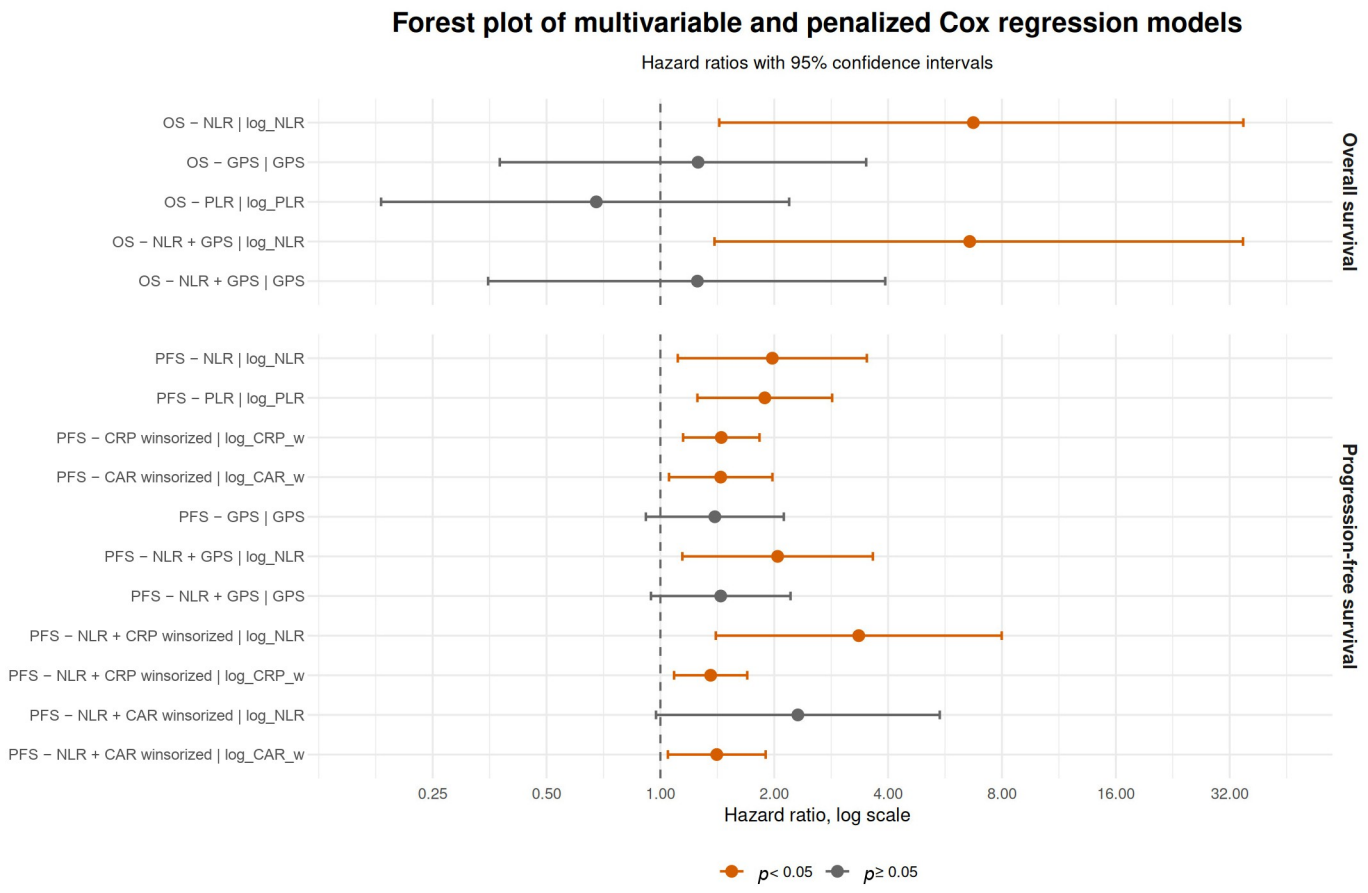
Forest plot of multivariable and penalized Cox regression models.

Elevated NLR demonstrated the most consistent prognostic effect across all Cox regression specifications (Table 5 and Figure 3). In univariable analysis, higher log-transformed NLR was associated with significantly shorter progression-free survival (HR 1.98, 95% CI 1.11–3.51, *p* = 0.020), and the association persisted after adjustment for GPS and acute-phase inflammatory markers.

The strongest association with progression risk was observed in the combined NLR + CRP model, in which elevated NLR remained independently associated with adverse PFS outcomes (HR 3.35, 95% CI 1.40–7.99, *p* = 0.007). Importantly, CRP also retained independent prognostic significance within this model, suggesting partially complementary inflammatory information.

PLR demonstrated significant prognostic value in univariable analysis (HR 1.89, 95% CI 1.25–2.85, *p* = 0.002), although its effect appeared less stable across penalized and multivariable approaches. Similarly, winsorized CRP and CAR retained significant associations with shorter PFS, although with more moderate effect sizes compared with NLR.

In contrast, GPS did not achieve statistical significance in either univariable or combined models, despite a consistent trend toward poorer outcomes with increasing score values.

For overall survival, elevated NLR remained associated with increased mortality risk in Firth penalized Cox regression models. However, confidence intervals were substantially wider in OS analyses, reflecting reduced precision due to the limited number of death events within the cohort.

To address major clinicopathologic confounding, a parsimonious multivariable Cox model stratified by ECOG performance status was constructed, adjusting for age, metastatic burden, tumor sidedness, and anti-VEGF exposure.

Within the adjusted model, NLR was no longer independently associated with progression-free survival (HR 1.41, 95% CI 0.81–2.43, *p* = 0.221) (Table 6). Increased metastatic burden remained significantly associated with adverse progression-free survival, while tumor sidedness also retained prognostic significance. The proportional-hazards assumption was satisfied in the final stratified model (global Schoenfeld test *p* = 0.358).

Time-dependent ROC analysis was performed to evaluate the discriminatory performance of NLR for survival outcomes across different follow-up intervals (Table 7). For progression-free survival, NLR demonstrated limited predictive discrimination, with AUC values remaining below 0.50 at 12, 24, and 36 months.

On the other hand, the estimated AUC for overall survival at 24 months was markedly higher (AUC = 0.944), suggesting high discriminatory performance. However, this result should be interpreted with caution due to the limited number of OS events observed in the cohort. With only 12 death events available for analysis, time-dependent ROC estimates may become unstable and potentially overestimate predictive performance. Consequently, the OS ROC findings should be considered exploratory rather than definitive.

LASSO penalized Cox regression was performed to explore variable selection and reduce potential overfitting in the progression-free survival models (Table 8). After shrinkage penalization, PLR, CAR, and GPS retained non-zero coefficients and were therefore selected within the final penalized model. In contrast, NLR and CRP coefficients were reduced to zero, suggesting that part of their prognostic information may overlap with other inflammatory biomarkers included in the model.

These findings indicate the presence of partial redundancy among systemic inflammatory indices, which is biologically plausible given that several markers reflect related inflammatory and nutritional pathways. The retention of PLR, CAR, and GPS after penalization suggests that these variables may capture complementary prognostic information in metastatic colorectal cancer.

**Figure 4:**
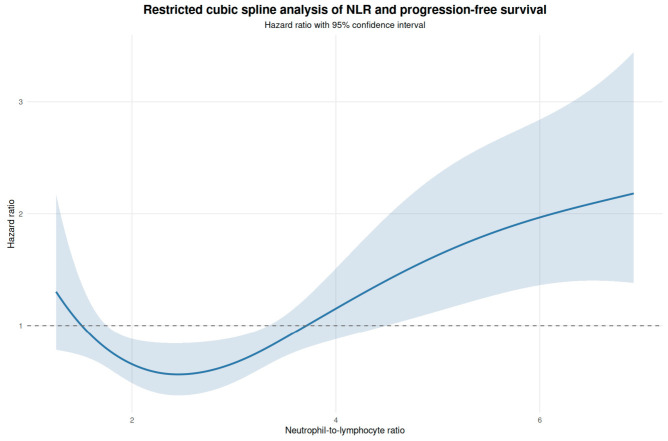
Restricted cubic spline analysis.

Restricted cubic spline modeling demonstrated that the association between NLR and progression-free survival was significant overall (χ^2^ = 14.30, *p* = 0.0025). The nonlinear component also reached statistical significance (χ^2^ = 6.13, *p* = 0.0467), indicating that the relationship between NLR and progression risk was not strictly linear across the biomarker spectrum (Figure 4).

Restricted cubic spline analysis demonstrated a nonlinear association between NLR and progression-free survival (Figure 4). The estimated hazard ratio initially decreased at lower NLR values, reaching the lowest risk around NLR values of approximately 2–3, after which the hazard progressively increased with rising NLR levels.

Beyond an NLR threshold of roughly 3.5–4, the risk of progression increased steadily, suggesting that elevated systemic inflammatory burden is associated with substantially poorer progression-free survival. At higher NLR values, the hazard ratio exceeded 2, indicating more than a twofold increase in progression risk compared with the reference region.

The widening confidence intervals observed at extreme NLR values likely reflect reduced patient density in these ranges and therefore greater statistical uncertainty. Nevertheless, the overall spline model and nonlinear component were statistically significant, supporting the presence of a non-proportional and nonlinear relationship between NLR and progression risk.

Sensitivity analysis using multiple imputation demonstrated persistence of the association between elevated NLR and shorter progression-free survival (Table 9). After imputation of missing values for inflammatory biomarkers, log-transformed NLR remained significantly associated with increased progression risk (HR 2.04, 95% CI 1.13–3.68, *p* = 0.018).

GPS did not retain statistical significance after multiple imputation, although a trend toward poorer progression-free survival with increasing GPS values was still observed (HR 1.44, 95% CI 0.94–2.22, *p* = 0.094). Overall, these findings suggest that the prognostic effect of NLR is relatively stable across different analytical approaches and handling strategies for missing data.

Bootstrap internal validation demonstrated moderate-to-good stability of the optimized inflammatory biomarker cut-off values across 500 resampling iterations (Table 10). Among the evaluated biomarkers, NLR showed a median bootstrap-derived cut-off of 2.75, with an interquartile range extending from 2.46 to 4.15 and a relatively narrow 95% bootstrap interval (1.51–4.32). These findings suggest acceptable reproducibility of the NLR threshold despite moderate variability across resampled datasets.

PLR demonstrated comparatively greater variability, although the median bootstrap-derived cut-off remained highly consistent at 122.96 across most iterations. The wider upper bootstrap confidence interval observed for PLR indicates reduced stability at higher threshold values.

CRP and CAR demonstrated relatively stable bootstrap-derived thresholds. For CRP, the median optimal cut-off remained fixed at 20.78 across the interquartile range, while CAR demonstrated a median cut-off of 5.40 with moderate dispersion.

A composite inflammatory score derived from selected biomarkers demonstrated significant prognostic performance for progression-free survival (Table 11). The reproducible composite inflammatory score demonstrated only modest prognostic performance for progression-free survival. In Cox regression analysis, higher composite inflammatory scores were associated with a trend toward shorter progression-free survival (HR 1.09, 95% CI 1.00–1.19, *p* = 0.064). The apparent concordance index was 0.572, and bootstrap validation yielded a mean C-index of 0.573 (95% bootstrap interval 0.480–0.669), indicating limited discriminatory performance.

## 4. Discussion

This retrospective cohort study evaluated the prognostic relevance of routinely available systemic inflammatory biomarkers in patients with metastatic colorectal cancer. The main finding was that systemic inflammation was consistently associated with adverse progression-related outcomes, although the strength and stability of these associations varied across biomarkers and modeling strategies. Among the evaluated markers, NLR showed the most reproducible prognostic signal, whereas PLR, CRP, CAR, and GPS appeared to capture partially overlapping but biologically distinct components of the systemic inflammatory response.

In the present analysis, CRP and PLR showed the strongest inverse correlations with PFS, suggesting that both acute-phase inflammation and platelet-related inflammatory activity are related to earlier disease progression. This observation aligns with literature data that CRP is an acute-phase protein synthesized by hepatocytes under the control of pro-inflammatory cytokines, particularly IL-6 (as well as IL-1 and TNF-α), and that elevated CRP reflects both tumor-associated inflammation and the broader systemic host response [16,19]. Within this framework, the CRP-to-albumin ratio (CAR) provides an integrated measure of systemic inflammation and nutritional status, because albumin is suppressed by the same cytokine milieu that induces CRP, thereby allowing CAR to more accurately capture the severity of cancer-related inflammation than either parameter alone [16,20,21]. The combination of these pathophysiological insights with the present findings supports a biologically plausible role for CRP as markers of aggressive disease biology and poorer progression-free outcomes in metastatic colorectal cancer.

NLR emerged as the most consistent marker across the survival analyses. Higher NLR was significantly associated with shorter PFS in Cox regression, remained relevant after adjustment for GPS and acute-phase inflammatory markers, and was also associated with OS in Firth penalized models. An important observation was that the strongest PFS association was found in the combined NLR + CRP model, where both NLR and winsorized CRP retained statistical significance. However, the apparent prognostic effect of NLR became attenuated after adjustment for major clinicopathologic confounders.

A systematic review and meta-analysis which included 71 studies and a total of 32,788 patients with colorectal cancer, evaluated the prognostic value of NLR for OS and surrogate endpoints [22]. The analysis showed that elevated NLR was consistently associated with shorter overall survival and poorer surrogate survival outcomes in both univariate and multivariate models [22]. After correction for publication bias in the subset of multivariate analyses, the pooled HR was estimated at 1.57 for OS and 1.38 for surrogate endpoints, indicating a significantly increased risk of death and disease recurrence or progression among patients with elevated NLR. The clinical relevance of NLR remained robust even after adjustment for other prognostic factors.

Another meta-analysis, which included 16 studies, evaluated the impact of elevated pre-treatment NLR values on survival in patients with colorectal cancer [23]. This synthesis demonstrated that increased NLR before treatment initiation was associated with significantly poorer overall survival (HR 1.81) and shorter progression-free survival (HR 2.10) [23].

In the retrospective analysis of the TRIBE study, which included patients with metastatic colorectal cancer treated with FOLFOXIRI or FOLFIRI in combination with bevacizumab, the impact of baseline NLR on survival was evaluated [24]. An NLR value ≥ 3 at baseline was associated with shorter PFS and OS in univariate analyses. In multivariate Cox models adjusted for major clinical and prognostic factors, including age, performance status, tumor burden, tumor sidedness, and other relevant covariates, NLR remained a significant predictor of OS, with an HR of 1.44 (95% CI 1.14–1.82) [24].

In the randomized ITACa trial, which investigated the role of inflammatory markers in patients with metastatic colorectal cancer treated with chemotherapy, including bevacizumab-containing regimens, joint models were used to integrate the longitudinal dynamics of NLR with survival analysis [25]. Changes in NLR over time had a strong impact on PFS: a one-unit increase in the natural logarithm of NLR (lnNLR) was associated with an HR of approximately 4.0 for progression, indicating that dynamic changes in systemic inflammation may have major consequences for tumor progression risk. In addition, baseline NLR showed adverse prognostic value in the subgroup treated with chemotherapy plus bevacizumab, independently of other clinical factors included in the model, suggesting that pre-treatment inflammatory status may influence the benefit derived from anti-angiogenic therapy [25].

PLR also demonstrated prognostic value for PFS, although its effect appeared less stable across advanced modeling approaches. A meta-analysis evaluating the prognostic value of the PLR in patients with colorectal cancer receiving chemotherapy included 19 studies (26 comparative cohorts), comprising a total of 4422 patients [26]. The pooled analysis demonstrated that elevated PLR values were associated with significantly shorter OS and PFS in patients with colorectal cancer undergoing chemotherapy (OS: HR 1.18, 95% CI 1.03–1.35, *p* = 0.02; PFS: HR 1.28, 95% CI 1.03–1.60, *p* = 0.03) [26]. However, sensitivity analysis for PFS revealed substantial fragility of the result: after exclusion of three studies, the association between PLR and PFS changed from statistically significant to non-significant, whereas the OS findings remained stable. The authors emphasized that the predictive value of PLR for PFS appears unstable and requires confirmation in additional studies [26].

An earlier meta-analysis including 27 studies and 13,330 patients evaluated the association between PLR and multiple survival endpoints in colorectal cancer [27]. The results showed that elevated PLR was associated with significantly shorter overall survival (OS: HR 1.40, 95% CI 1.21–1.62, *p* < 0.00001), disease-free survival (DFS: HR 1.44, 95% CI 1.09–1.90, *p* = 0.01), and recurrence-free survival (RFS: HR 1.48, 95% CI 1.13–1.94, *p* = 0.005) [27]. In contrast, no significant association was observed between PLR and progression-free survival (PFS: HR 1.14, 95% CI 0.84–1.54, *p* = 0.40) or cancer-specific survival (CSS: HR 1.16, 95% CI 0.88–1.53, *p* = 0.28) [27]. This pattern further supports the notion that the effect of PLR on PFS is weaker.

In a large observational study including 1314 patients who underwent surgery for colorectal cancer, preoperative PLR was investigated as a prognostic biomarker [28]. In univariate analyses, patients in the highest PLR quintile had a 70% higher risk of death and a 52% higher risk of recurrence compared with those in the lowest quintile (OS: HR 1.701, 95% CI 1.267–2.282, *p* < 0.001; DFS: HR 1.522, 95% CI 1.114–2.080, *p* = 0.008). In multivariable Cox regression, PLR remained an independent predictor of OS (HR 1.511, 95% CI 1.103–2.070, *p* = 0.010) [28].

A separate meta-analysis focused on the prognostic role of PLR and the LMR in colorectal cancer included 19 studies evaluating the association between PLR and OS and 8 studies assessing the relationship with DFS [29]. Elevated PLR values were associated with an increased risk of death (OS: HR 1.23, 95% CI 1.04–1.44, *p* = 0.01), indicating a modest but statistically significant adverse effect on overall survival [29].

GPS did not reach statistical significance in most models, despite a consistent trend toward poorer outcomes. This may be explained by the low baseline distribution of GPS in this cohort, which reduced discriminatory variability. In a meta-analysis that investigated the prognostic value of GPS/mGPS in colorectal cancer, the authors noted that in several included studies the hazard ratios from multivariable models for GPS/mGPS were less statistically significant than those for other systemic inflammatory markers, such as PLR, NLR, or similar indices [30]. These competing markers sometimes retained significance while GPS/mGPS did not, so the contribution of GPS/mGPS became non-significant in certain adjusted models, despite overall pooled results supporting GPS/mGPS as a prognostic indicator for OS and CSS in CRC [30].

In patients with advanced cancers treated with immune checkpoint inhibitors, a separate meta-analysis showed that higher GPS (scores 1–2) was associated with worse PFS overall (HR 1.48, 95% CI 1.03–2.13) [31]. However, the prognostic effect on PFS was not uniform across subgroups. In cancer-type-specific analyses, elevated GPS did not reach statistical significance for PFS in non-small cell lung cancer and gastric cancer, even though the pooled hazard ratios pointed toward increased risk (e.g., non-small cell lung cancer HR 1.63, 95% CI 0.95–2.78) [31].

In a development and validation study of an “improved GPS” (iGPS) in stage 0–III CRC after curative resection, the conventional GPS was associated with RFS in the training set but failed to remain an independent predictor of RFS in the validation cohort (HR 1.817, 95% CI 0.962–3.432; *p* = 0.066) [32]. On the other hand, the iGPS, based on optimized cut-offs for CRP and albumin, was an independent prognostic factor for RFS in the validation set (HR 2.273, 95% CI 1.212–4.264; *p* = 0.011) and showed higher C-indices for both RFS and OS than the conventional GPS [32]. These findings suggest that, particularly in early-stage, surgically treated cohorts with relatively low event rates, the score specification and distribution of CRP/albumin values can limit the discriminative power of the conventional GPS, while refined scoring (iGPS) may better capture prognostic information [32].

The nonlinear spline analysis adds an important methodological and biological layer to the findings. The association between NLR and PFS was not strictly linear: risk appeared lowest at intermediate NLR values and increased progressively above approximately 3.5–4.0. This observation may help explain why published NLR cut-offs vary widely across studies. A single dichotomous threshold may oversimplify a more complex relationship between systemic inflammation and tumor progression. In this context, restricted cubic spline modeling provides a more nuanced interpretation than conventional linear or binary analyses.

In a cohort study of 195 patients with stage II–III and IV colorectal cancer, the preoperative NLR was evaluated as a prognostic and predictive biomarker [33]. Using ROC curve analysis, the optimal NLR cut-off was identified as 3. Patients were stratified into a high-NLR group (NLR ≥ 3) and a low-NLR group (NLR < 3). High-NLR patients experienced significantly shorter OS and PFS, and also had a lower overall response rate to treatment. In multivariable Cox models, elevated NLR remained strongly associated with poorer PFS (HR 3.17; 95% CI 2.12–4.75), indicating that a preoperative NLR ≥ 3 identifies a subgroup at substantially higher risk of progression and death [33].

A very large retrospective cohort from the Chang Gung Research Database included 16,990 patients who underwent surgery for colorectal cancer and had preoperative blood counts available [34]. An optimal NLR cut-off of 3.59 was calculated using a dedicated macro based on time-to-event statistics. Patients with NLR ≥ 3.59 had markedly worse 5-year DFS and OS than those with lower NLR. In univariate analyses, 5-year DFS was 44.7% versus 68.1%, and 5-year OS 50.1% versus 73.6% in high- versus low-NLR groups. After adjustment for age, sex, BMI, hemoglobin, hypoalbuminaemia, CEA, PLR, TNM stage and chemotherapy, high NLR remained an independent predictor of poor outcome (HR 1.319 for DFS and 1.611 for OS; both *p* < 0.0001) [34].

In a separate postoperative series of 788 patients undergoing curative colorectal cancer surgery, NLR was measured preoperatively and again 1 year after surgery [35]. A post-operative cut-off of 3.3 was applied to the 1-year NLR. Patients were classified into four trajectories (low–low, low–high, high–low, high–high). Median postoperative NLR values were significantly lower than preoperative values (2.8 vs. 4.1), but a post-NLR > 3.3 at 1 year was associated with substantially worse survival and higher recurrence. The “low–high” group (normal pre-, elevated post-NLR) showed the poorest prognosis, with a 5-year survival of 42.6% compared with 79.8% in the low–low group. Multivariate Cox analysis confirmed post-NLR > 3.3 as an independent predictor of adverse outcome (HR 3.49; 95% CI 2.41–5.04) [35].

Support for an “optimal interval” of NLR, with risk increasing beyond a threshold, comes from a large multicentre cohort of 2612 patients with cancer cachexia from various primary tumours [36]. Using restricted cubic spline modelling in Cox regression, the relationship between NLR and all-cause mortality showed an inverted L-shaped dose–response curve. The optimal cut-off for mortality prediction in cachectic cancer patients was an NLR of 3.5; values ≥ 3.5 were independently associated with higher mortality (HR 1.51; 95% CI 1.33–1.71), even after adjustment for extensive clinical and laboratory covariates [36]. When NLR was modelled in quartiles, increasing quartiles were associated with progressively worse prognosis, and sensitivity analyses using classic cancer thresholds (NLR <3 vs. ≥3; and <3, 3–5, ≥5) yielded similar trends: intermediate (3–5) and high (≥5) NLR groups had significantly increased hazards compared with NLR < 3 [36]. These data biologically support the concept of lowest risk at intermediate NLR values and a clear, monotonic rise in risk once NLR surpasses roughly 3–3.5.

Consistent with this, multiple colorectal cancer studies have defined “high NLR” in the range of approximately 3–5 and demonstrated marked increases in recurrence or progression risk above those cut-offs. For example, in non-metastatic CRC, a cut-off of 3 identified patients with significantly poorer DFS after curative surgery, independently of other clinicopathological factors [37]. In a large series of stages I–III CRC, NLR > 3 predicted worse 5-year DFS, particularly in colon cancer [38], and in a curative surgery cohort, NLR > 4.7 was a strong adverse factor, especially in stage II disease [39].

The LASSO analysis further highlighted the redundancy among inflammatory biomarkers. After penalization, PLR, CAR, and GPS retained non-zero coefficients, while NLR and CRP were reduced toward zero. The prognostic behavior of NLR appeared heterogeneous across analytical frameworks. Although NLR demonstrated associations with progression-free survival in several univariable and partially adjusted analyses, its prognostic effect became attenuated after clinicopathologic adjustment and was inconsistently retained in penalized regression models. Moreover, time-dependent ROC analyses demonstrated limited standalone discriminatory performance for progression-free survival. These findings suggest that NLR may capture only part of a broader inflammatory and disease-burden signal rather than functioning as a universally robust independent prognostic biomarker.

The internally validated composite inflammatory score showed better discrimination than the NLR + GPS model, with an optimism-corrected C-index of approximately 0.72. This finding is clinically relevant because it suggests that combining inflammatory and nutritional dimensions may improve risk stratification. In stage IV colorectal cancer, several studies have evaluated composite inflammatory–nutritional scores as prognostic markers, with particular focus on CONUT, PNI and mGPS, and on PNI alone in metastatic disease.

In a large retrospective cohort of 996 patients with stage IV CRC treated at a single cancer center, three host-related indices were assessed at diagnosis: the Controlling Nutritional Status (CONUT) score, the Prognostic Nutritional Index (PNI), and the modified Glasgow Prognostic Score (mGPS) [40]. After adjustment, all three measures were independent prognostic factors for OS (all *p* < 0.001). CONUT (low 0–1, intermediate 2–3, high ≥ 4) showed stepwise separation of survival: median OS 30.3 vs. 23.3 vs. 16.6 months, with significant hazard increments between each adjacent category (e.g., high vs. low: HR 1.57, 95% CI 1.23–1.98) [40]. PNI < 48.0 identified patients with shorter OS (19.8 vs. 33.8 months; adjusted HR 1.39, 95% CI 1.19–1.62). mGPS 1–2 was also associated with progressively worse OS versus mGPS 0, but there was no significant difference between mGPS 1 and 2 (HR 1.12, 95% CI 0.88–1.41), indicating less fine stratification [40].

In newly diagnosed metastatic CRC, a retrospective study of 308 patients evaluated baseline PNI as an immuno-nutritional marker alongside neutrophil-to-lymphocyte ratio (NLR) and platelet-to-lymphocyte ratio (PLR) [41]. Using ROC analysis, an optimal PNI cut-off of 46 was defined; 182 patients (59%) had PNI > 46 (PNI-High) and 126 (41%) had PNI ≤ 46 (PNI-Low) 2. Median OS was significantly longer in the PNI-High group (28.4 vs. 19.1 months, *p* < 0.001) [41]. In multivariable Cox regression, a high PNI remained an independent favorable prognostic factor for OS (HR 0.61, 95% CI 0.42–0.87, *p* = 0.007), whereas NLR and PLR, although associated with outcome in univariate analyses, lost statistical significance after adjustment for other clinical variables [41].

Similarly, a more recent single-center study of 253 patients with metastatic CRC examined the prognostic value of PNI at diagnosis using the classical Onodera formula [42]. The mean PNI was 46.6, which was adopted as the cut-off to define low (<46.6) and high (≥46.6) PNI groups 3. Patients with PNI ≥ 46.6 had significantly longer OS than those with lower values (53.06 vs. 38.80 months, *p* = 0.039) [42]. PFS was numerically better in the high-PNI group (PFS not reached vs. 25.66 months), though this difference did not reach statistical significance (*p* = 0.265) [42]. Kaplan–Meier curves showed a clear separation of survival trajectories between PNI strata, including within right- and left-sided primary tumor subgroups and across major metastatic sites (liver, lung, lymph nodes) [42]. The authors emphasize that PNI, as a simple and inexpensive composite of albumin and lymphocyte count, can be easily applied as a prognostic marker in metastatic CRC and reflects relevant aspects of host immune and nutritional status that are not fully captured by tumor-centred variables [42].

The time-dependent ROC analysis showed limited discrimination of NLR alone for PFS, despite its significant association with outcome in Cox regression. This distinction is important. A biomarker may be statistically associated with survival without being sufficiently accurate as a standalone classifier. Therefore, NLR should not be interpreted as an isolated predictive tool, but rather as one component of a broader prognostic framework. The high AUC observed for OS at 24 months should be considered exploratory because of the small number of death events and the risk of unstable estimation.

The study also has limitations. Its retrospective design introduces the possibility of selection bias and residual confounding. The single-center setting and the relatively low number of patients limits generalizability, and external validation was not available. OS analyses were limited by the small number of death events, resulting in wide confidence intervals even after Firth penalization. Missing data for CRP, albumin, and CAR may also have influenced some estimates, although multiple imputation supported the persistence of the NLR effect.

## 5. Conclusions

NLR showed the most consistent association with adverse outcomes, while PLR, CRP, CAR, and GPS contributed additional but partially overlapping prognostic information.

The nonlinear relationship between NLR and PFS suggests that inflammatory risk may not be adequately captured by simple linear assumptions.

Although isolated biomarkers demonstrated limited standalone discrimination, the internally validated composite inflammatory score showed encouraging performance.

Future multicenter studies should externally validate these findings and integrate inflammatory biomarkers with molecular, radiological, and treatment-related variables to develop clinically applicable prognostic models.

## Figures and Tables

**Table 1:** Descriptive statistics of inflammatory biomarkers.

Variable	Mean ± SD	Median (IQR)	Range
Albumin	3.96 ± 0.74	4.11 (3.71–4.36)	1.42–4.80
CAR	4.59 ± 6.87	1.44 (0.39–4.72)	0.04–26.99
CRP	17.70 ± 28.29	5.01 (1.63–20.78)	0.21–122.00
GPS	0.40 ± 0.56	0 (0–1)	0–2
NLR	3.22 ± 1.68	3.20 (1.80–4.15)	1.24–8.28
PLR	211.74 ± 119.50	188.00 (122.96–256.30)	73.45–602.90

Legend: CAR = C-reactive protein-to-albumin ratio; CRP = C-reactive protein; GPS = Glasgow Prognostic Score; NLR = neutrophil-to-lymphocyte ratio; PLR = platelet-to-lymphocyte ratio; SD = standard deviation; IQR = interquartile range.

**Table 2:** Correlation analysis between inflammatory biomarkers and survival outcomes.

Marker	Outcome	Pearson r	Pearson *p*-Value	Spearman ρ	Spearman *p*-Value
NLR	PFS	−0.145	0.078	−0.110	0.184
NLR	OS	−0.207	0.011	−0.125	0.130
PLR	PFS	−0.227	0.006	−0.185	0.024
PLR	OS	−0.120	0.145	−0.080	0.335
CRP	PFS	−0.245	0.016	−0.251	0.014
CRP	OS	−0.134	0.193	−0.078	0.448
CAR	PFS	−0.192	0.082	−0.142	0.199
CAR	OS	−0.056	0.616	0.097	0.384
GPS	PFS	−0.148	0.072	−0.184	0.025
GPS	OS	−0.232	0.005	−0.205	0.012

Legend: CAR = C-reactive protein-to-albumin ratio; CRP = C-reactive protein; GPS = Glasgow Prognostic Score; NLR = neutrophil-to-lymphocyte ratio; PLR = platelet-to-lymphocyte ratio; PFS = progression-free survival; OS = overall survival.

**Table 3:** Optimal cut-off values and Kaplan–Meier survival analysis for progression-free survival.

Marker	Outcome	Optimal Cut-Off	χ^2^	*p*-Value	FDR-Adjusted *p*-Value
NLR	PFS	4.15	9.46	0.002	0.003
PLR	PFS	122.96	18.64	<0.001	<0.001
CRP	PFS	20.78	19.33	<0.001	<0.001
CAR	PFS	5.40	18.38	<0.001	<0.001
GPS	PFS	0	0.29	0.593	0.659

Legend: CAR = C-reactive protein-to-albumin ratio; CRP = C-reactive protein; GPS = Glasgow Prognostic Score; NLR = neutrophil-to-lymphocyte ratio; PLR = platelet-to-lymphocyte ratio; PFS = progression-free survival; FDR = false discovery rate.

**Table 4:** Stabilized Cox proportional hazards models for progression-free survival.

Model	Predictor	HR (95% CI)	*p*-Value
PFS − NLR	log_NLR	1.98 (1.11–3.51)	0.020
PFS − PLR	log_PLR	1.89 (1.25–2.85)	0.002
PFS − CRP winsorized	log_CRP_w	1.45 (1.15–1.83)	0.002
PFS − CAR winsorized	log_CAR_w	1.44 (1.05–1.98)	0.022
PFS − GPS	GPS	1.39 (0.92–2.12)	0.122

Legend: HR = hazard ratio; CI = confidence interval; CAR = C-reactive protein-to-albumin ratio; CRP = C-reactive protein; GPS = Glasgow Prognostic Score; NLR = neutrophil-to-lymphocyte ratio; PLR = platelet-to-lymphocyte ratio; PFS = progression-free survival.

**Table 5:** Multivariable and penalized Cox regression models for progression-free and overall survival.

Model	Predictor	HR (95% CI)	*p*-Value	Method
PFS − NLR	log_NLR	1.98 (1.11–3.51)	0.020	Cox proportional hazards
PFS − PLR	log_PLR	1.89 (1.25–2.85)	0.002	Cox proportional hazards
PFS − CRP winsorized	log_CRP_w	1.45 (1.15–1.83)	0.002	Cox proportional hazards
PFS − CAR winsorized	log_CAR_w	1.44 (1.05–1.98)	0.022	Cox proportional hazards
PFS − GPS	GPS	1.39 (0.92–2.12)	0.122	Cox proportional hazards
PFS − NLR + GPS	log_NLR	2.04 (1.14–3.65)	0.016	Cox proportional hazards
PFS − NLR + GPS	GPS	1.44 (0.94–2.21)	0.090	Cox proportional hazards
PFS − NLR + CRP winsorized	log_NLR	3.35 (1.40–7.99)	0.007	Cox proportional hazards
PFS − NLR + CRP winsorized	log_CRP_w	1.36 (1.09–1.70)	0.007	Cox proportional hazards
PFS − NLR + CAR winsorized	log_NLR	2.31 (0.97–5.48)	0.058	Cox proportional hazards
PFS − NLR + CAR winsorized	log_CAR_w	1.41 (1.05–1.90)	0.024	Cox proportional hazards
OS − NLR	log_NLR	6.72 (1.43–34.78)	0.016	Firth penalized Cox
OS − GPS	GPS	1.26 (0.38–3.50)	0.685	Firth penalized Cox
OS − PLR	log_PLR	0.68 (0.18–2.19)	0.526	Firth penalized Cox
OS − NLR + GPS	log_NLR	6.57 (1.39–34.70)	0.017	Firth penalized Cox
OS − NLR + GPS	GPS	1.25 (0.35–3.93)	0.711	Firth penalized Cox

Legend: HR = hazard ratio; CI = confidence interval; PFS = progression-free survival; OS = overall survival; NLR = neutrophil-to-lymphocyte ratio; PLR = platelet-to-lymphocyte ratio; CRP = C-reactive protein; CAR = C-reactive protein-to-albumin ratio; GPS = Glasgow Prognostic Score.

**Table 6:** Parsimonious multivariable Cox regression model for progression-free survival adjusted for major clinicopathologic confounders.

Variable	HR (95% CI)	*p*-Value
log_NLR	1.41 (0.81–2.43)	0.221
Age	1.00 (0.97–1.02)	0.765
Metastatic burden	0.80 (0.66–0.96)	0.019
Right-sided tumor	0.35 (0.21–0.59)	<0.001
Anti-VEGF exposure	0.90 (0.58–1.40)	0.634

Legend: HR = hazard ratio; CI = confidence interval; NLR = neutrophil-to-lymphocyte ratio; VEGF = vascular endothelial growth factor.

**Table 7:** Time-dependent ROC analysis for NLR.

Outcome	Marker	Time Point (Months)	AUC
PFS	NLR	12	0.480
PFS	NLR	24	0.461
PFS	NLR	36	0.459
OS	NLR	24	0.944

Legend: ROC = receiver operating characteristic; AUC = area under the curve; NLR = neutrophil-to-lymphocyte ratio; PFS = progression-free survival; OS = overall survival.

**Table 8:** LASSO penalized Cox regression for progression-free survival.

Predictor	Coefficient	Selected
log_NLR	0.000	No
log_PLR	0.511	Yes
log_CRP_w	0.000	No
log_CAR_w	0.149	Yes
GPS	0.331	Yes

Legend: NLR = neutrophil-to-lymphocyte ratio; PLR = platelet-to-lymphocyte ratio; CRP = C-reactive protein; CAR = C-reactive protein-to-albumin ratio; GPS = Glasgow Prognostic Score.

**Table 9:** Cox regression model after multiple imputation.

Predictor	HR	95% CI	*p*-Value
log1p(NLR)	2.04	1.13–3.68	0.018
GPS	1.44	0.94–2.22	0.094

Legend: HR = hazard ratio; CI = confidence interval; NLR = neutrophil-to-lymphocyte ratio; GPS = Glasgow Prognostic Score -lymphocyte.

**Table 10:** Bootstrap validation of optimal biomarker cut-off values.

Marker	Bootstrap Iterations	Median Cut-Off	IQR	95% Bootstrap Interval	Mean Cut-Off ± SD
NLR	500	2.75	2.46–4.15	1.51–4.32	3.20 ± 0.99
PLR	500	122.96	122.96–122.96	122.96–234.51	132.48 ± 29.93
CRP	500	20.78	20.78–20.78	1.63–20.78	17.51 ± 6.65
CAR	500	5.40	4.04–5.40	1.22–5.40	4.56 ± 1.54

Legend: NLR = neutrophil-to-lymphocyte ratio; PLR = platelet-to-lymphocyte ratio; CRP = C-reactive protein; CAR = C-reactive protein-to-albumin ratio; SD = standard deviation; IQR = interquartile range.

**Table 11:** Composite inflammatory score model and bootstrap validation.

Parameter	Value
Formula	z(log_NLR) + z(log_PLR) + z(log_CRP) + z(log_CAR) + z(GPS)
Patients included	83
PFS events	59
HR	1.09
95% CI	1.00–1.19
*p*-value	0.064
C-index	0.572
Bootstrap mean C-index	0.573
95% bootstrap interval	0.480–0.669
Bootstrap iterations	500

Legend: CI = confidence interval; C-index = concordance index; NLR = neutrophil-to-lymphocyte ratio; PLR = platelet-to-lymphocyte ratio; CRP = C-reactive protein; CAR = C-reactive protein-to-albumin ratio; HR = hazard ratio; GPS = Glasgow Prognostic Score -lymphocyte.

## Data Availability

The data presented in this study are available on request from the corresponding author. The data are not publicly available due to local regulations.

## Abbreviations

The following abbreviations are used in this manuscript:

AUC	Area under the curve
CAR	C-reactive protein-to-albumin ratio
CI	Confidence interval
CRC	Colorectal cancer
CRP	C-reactive protein
DFS	Disease-free survival
FDR	False discovery rate
GNRI	Geriatric nutrition risk index
GPS	Glasgow Prognostic Score
HR	Hazard ratio
IBI	Inflammatory burden index
IL-8	Interleukin-8
IQR	Interquartile range
LASSO	Least Absolute Shrinkage and Selection Operator
LMR	Lymphocyte-to-monocyte ratio
LMS	Lymphocyte-monocyte score
mCRC	Metastatic colorectal cancer
mGPS	Modified Glasgow Prognostic Score
MICE	Multiple imputation by chained equations
NLR	Neutrophil-to-lymphocyte ratio
NLS	Neutrophil-lymphocyte score
NPS	Neutrophil-platelet score
OS	Overall survival
PFS	Progression-free survival
PIV-PNI	Pan-immune-inflammation value–prognostic nutritional index
PLR	Platelet-to-lymphocyte ratio
PLS	Platelet-lymphocyte score
PNI	Prognostic nutritional index
RCS	Restricted cubic spline
ROC	Receiver operating characteristic
SD	Standard deviation
SII	Systemic immune-inflammation index
TNM	Tumor, node, metastasis
